# Attitude, Knowledge, and Practice on Evidence-Based Nursing among Registered Nurses in Traditional Chinese Medicine Hospitals: A Multiple Center Cross-Sectional Survey in China

**DOI:** 10.1155/2016/5478086

**Published:** 2016-07-27

**Authors:** Fen Zhou, Yufang Hao, Hong Guo, Hongxia Liu

**Affiliations:** ^1^Department of Clinical Nursing, Nursing School, Beijing University of Chinese Medicine, 11 North Third Road, Chaoyang District, Beijing 100029, China; ^2^Joanna Briggs Institute Center of Excellence, Nursing School, Beijing University of Chinese Medicine, 11 North Third Road, Chaoyang District, Beijing 100029, China; ^3^Nursing School, Beijing University of Chinese Medicine, 11 North Third Road, Chaoyang District, Beijing 100029, China; ^4^Center for Nursing Research, Nursing School, Beijing University of Chinese Medicine, 11 North Third Road, Chaoyang District, Beijing 100029, China

## Abstract

*Objective.* This study was to describe RNs' attitude, knowledge, and practice on evidence-based practice (EBP) in traditional Chinese nursing field and to estimate the related sociodemographic and professional factors.* Methods.* A multiple institutional cross-sectional survey design with self-reported EBP Questionnaire (EBPQ) and self-designed questionnaires were used.* Results.* The average scores of the total EBPQ were with a mean of 4.24 (SD = 0.79). The score of attitude was the highest one, followed by the knowledge score, and the lowest one is practice. RNs with longer experience reported stronger EBP knowledge (*H* = 6.64, *P* < 0.05). And RNs under higher working pressure reported less positive attitudes (*ρ* = 0.17, *P* < 0.001), whereas RNs holding negative professional attitude reported lower scores (Spearman's *ρ*: 0.12 to 0.15, *P* < 0.001). Significant statistics were found between RNs with research experience and without in attitude (*t* = −2.40, *P* < 0.05) and knowledge (*t* = −2.43, *P* < 0.05).* Conclusions.* Respondents generally viewed EBP positively and their attitudes towards EBP tended to be more positive than knowledge and practice of EBP. Data also showed that longer working experience, having administrative position, research experience, lighter working load, and better professional attitude might facilitate EBP.

## 1. Introduction

Due to the increasing internal and external expectations of higher quality nursing, it is no longer acceptable for nurses to deliver nursing care only on experience and textbook knowledge [1]. Clinical nurses are expected to systematically gather the best research evidence, draw from nursing experience, and consider patient's preferences when they are making professional decisions [2]. That approach is defined as evidence-based practice (EBP).

In western modern nursing field, EBP is evolving fast and has got some achievements. A great number of systematic reviews, evidence summaries, and practice guidelines could be searched on Cochrane Library and Joanna Briggs Institute Library. Evidence implementation and dissemination lag behind evidence synthesis and sometimes are met with resistance; however, they also proceed step by step. Some studies reported positive outcomes on clients and nurses after EBP. According to Vortherms et al., implementation of an evidence-based oncology outpatient staffing system increased working efficiency, reduced overtime, and improved patient and nurse satisfaction after a six-month pilot research [3]. Muhumuza et al. reported the best practice implementation improved healthcare worker hand hygiene even in a low-resource setting in Uganda [4]. Additionally, a cost-benefit analysis on Electronic Clinical Procedural Resource Supporting Evidence-Based Practice by Schoville et al. showed significant savings [5]. What is more, the EBP development increasingly emphasized the international collaboration and established several own nursing organizations. The first and largest EBP institution focusing mainly on nursing, the Joanna Briggs Institute (JBI), was established in 1996. Until now, it has over 70 entities across the world [6]. Another organization of influence on EBP in nursing is Registered Nurse Association of Ontario (RNAO) which developed more than 50 best practice guidelines and each of them was implemented in the real clinical nursing context [7].

Different from western nursing, traditional Chinese nursing (TCN) is based on traditional Chinese medicine (TCM) theory and fifteen unique nursing techniques, such as acupressure, scrapping, herbal bath, and TCM decoction preparing method [8]. Nowadays, TCN in China is very popular, so there are a great number of nurses working for TCN. According to the official data of the year 2009, there were 440.7 thousand different levels of medical service institutions providing TCN, and the data of the year 2011 showed that 1.86 million registered nurses (RN) worked in those institutions [9]. With the increasing acceptance of TCM in the western countries, nurses in the United States tend to disseminate TCM information and education to patients [10]. And some overseas nursing schools began to send students to China to learn TCN [11].

EBP in TCN, however, is less developed but is quickly gaining momentum. Initial retrieval on National Knowledge Infrastructure (CNKI, a common Chinese database) found that 1470 scientific citations were related to TCN, which also gives a hint that EBP in TCN field enjoyed a strong research base. On the other hand, the School of Nursing at Beijing University of Chinese Medicine in China was authorized as the JBI's subcenter in 2014 and then was regarded as one of the Best Practice Spotlight Organizations of RNAO in 2015. Both of these international events imply that the potential of EBP in TCN field could not be ignored. Although some developments were acquired, EBP in TCN field is still at the beginning stage.

Some research findings showed that changing the attitude and enhancing the knowledge of nurses are the first step in EBP [12]. McCleary and Brown conducted a study on 528 graduate nurses working in educational pediatric hospitals of Canada and reported that the nurses' knowledge of EBP and their positive attitude towards it will contribute to its implementation in healthcare system [13]. Melnyk et al. also stated that acquiring knowledge about research methods and having the skill to evaluate research reports critically may enable overcoming the obstacles hindering the application of research findings and thus will lead to improvement of healthcare quality [14]. Hence, the EBP attitude, knowledge, and skills of nurses are so important.

Just because of the awareness of the importance of this, there are some investigations that have been conducted to assess these aspects of nurses in other countries [15–19]. And the most common instrument used is the Evidence-Based Practice Questionnaire (EBPQ), which has been applied in 22 research studies and 5 educational and training purpose studies. Among them, 44% were conducted in the United States, 33% in Europe (only 2 in the United Kingdom), and 22% in other countries (Saudi Arabia, New Zealand, Australia, South Korea, and China) [20].

Unfortunately, no study has investigated the data regarding similar research in China in TCN field. Only a similar study by Yang and Tang was conducted 7 years ago to assess the perception and attitude of clinical nurses in China, but RNs are not from TCN field [21]. First, as we know, the situation might be changed during a period of 7 years. Second, compared with general hospitals in China, the nurses from TCM hospitals are with lower education background [22]. In addition, the ratio of doctors to nurses in TCM hospitals is surprisingly low, only 1 : 0.98 (in general hospitals, it is 1 : 1.28), and the ratio of patients to nurses is 1 : 0.39 (in general hospitals, it is 1 : 0.47) [23]. Because they enjoy the same level of professional autonomy, RNs from TCN hospitals might be busier.

Hence, it is worth conducting a survey in the same direction among RNs in TCM hospitals in China. Actually, we designed and finished an investigation program which contains two topics: (1) the status of RNs' barriers of research utilization (RU) and (2) the knowledge, attitude, and practice on EBP. Although several relations existed, the two topics are not the same. The major difference between them is that the RU is only a part of EBP. And the two topics have different emphasis points: the former one could help to explore more barriers due to nurses themselves and outside environment, while the latter one only evaluates RNs' self-conditioning on EBP. In 2015, part of our survey was published and it was found that RNs from TCM hospitals perceived different RU barriers compared with RNs from general hospitals [24]. For this paper, we are going to investigate the status of the latter topic of RNs and its influential factors, in order to comprehensively understand our participants.

## 2. Methods

This paper was prepared according to Strengthening the Reporting of Observational Studies in Epidemiology (STROBE) Statement [25].

### 2.1. Study Design and Sample Distribution

This is a cross-sectional survey which used a convenience sample of RNs employed in four TCM hospitals in the capital of China, Beijing. All the hospitals surveyed are tertiary-level teaching hospitals (which means the top level hospital certified by local healthcare government). RNs, in China, are different from those in western countries, who were defined as officially certified for clinical nursing practice by the local government regardless of their educational degree. All qualified RNs with undergraduate education from all shifts were included. Student nurses were excluded.

### 2.2. Ethical Approval

Ethical approval was obtained from the institutional review board of Beijing University of Chinese Medicine (approval number 2015BZHYLL0407). Each participant received a questionnaire with a paragraph describing the rationale for the study and informed verbal consent was obtained prior to the study.

### 2.3. Instrument Design

Instruments used in this survey included a self-designed questionnaire (which is the same as the published paper) and the validated Chinese version of the EBPQ [15].

The EBPQ was developed by D. Upton and P. Upton [15] in 2005 to assess the EBP knowledge, attitude, and practice among nurses. In 2009, Yang and Tang translated it into Chinese and reported that the Chinese version's Cronbach alpha value was 0.94 and the subscales' values were 0.79 to 0.94 [21]. This EBPQ is a self-completion questionnaire, composed of 24 items distributed among three subscales: knowledge/skills (14 items), attitude (4 items), and practice (6 items). Each item is scored on a scale of 1–7, with a higher score being associated with a more positive attitude towards EBP or use and knowledge of EBP. Responses of each item were considered positive if scores were greater than 4 [26]. Previous studies have assessed Cronbach's alpha coefficient of 0.87 for the entire questionnaire: 0.85 for the practice subscale, 0.79 for the attitudes subscale, and 0.91 for the knowledge/skills subscale [15]. In our pilot twenty-sample survey, Cronbach's alpha values were 0.88 and subscales' values were 0.80 to 0.91. For the data analysis, we applied both means of total and average EBPQ scores to report each subscale, in order to perform comparison with similar studies.

### 2.4. Data Analysis

The data were analyzed accordingly using the Statistical Package for the Social Sciences version 18.0 (SPSS Inc., Chicago, IL, USA). Mean, standard deviations, and cross tables were used in the description of variables. Independent sample* t*-test and nonparametric test were applied in the inferential statistical analysis when comparing groups in all variables. The relationships between self-perceived working pressure and attitude to nursing related to the EBPQ were analyzed by Spearman rank correlation. The cutoff for level of statistical significance was set at *P* < 0.05 and all tests were 2-sided.

The respondents were grouped as three subgroups depending on their different level of clinical experience (<10 years, 10~15 years, and >15 years) and first educational level (diploma, associate, bachelor, and higher degree). To simplify the results, some categories were grouped as “yes” and “no” answers, such as nursing administrator or not and research experience or not. Self-perceived working pressure and attitude to nursing (job satisfaction) were considered ordinal variables. For EBPQ, we applied both means of total and average scores to report each subscale, in order to perform comparison with similar studies.

## 3. Results

### 3.1. Respondents' Characteristics

950 questionnaires were disseminated; only 818 (86.11%) surveys were returned, of which 648 (79.22%) were completed. The majority of the participants were 35 years of age or younger, had practiced for 7 years or less, and were on managerial role and with neither like nor dislike attitude towards their nursing working. Almost half of them possessed diploma as their first educational background, perceived moderate working stress, and were without research experience. (The details of respondents' characteristics could be found in the previous published article [24].)

### 3.2. Knowledge of Attitudes to and Practice of EBP

The average scores of the total EBPQ ranged from 2.04 to 6.63, with a mean of 4.24 (SD = 0.79). The subscale of attitude was the highest one with mean of 4.75 (SD = 1.01), followed by the knowledge (mean = 4.17, SD = 0.84), and the lowest one is practice (mean = 4.08, SD = 1.05) (Table 1).

The items' scores of 1 to 4 were combined and represented as negative items. The lowest score and the most negative one for attitude was for the item “making time in a work schedule for research.” The mean score for this item was 3.84 out of 7, while the negative rate of it was 72.8%. And the lowest score for knowledge was “converting information needs into a research question”; the mean score was 3.76 out of 7. However, the most negative item of knowledge was “research skills,” with the highest negative rate in the whole scale being 83.8%. The item with the lowest practice score was “critically appraising literature.” The respondents' mean score for this item was 3.76 out of 7; it was also the most negative one in practice subscale, with 75.2% negative rate (Table 2).

### 3.3. Comparisons of Groups of Nurses

#### 3.3.1. Relationships

Spearman's correlations were calculated for attitude to nursing and self-perceived working pressure related to EBPQ. Although no correlation value (Spearman's *ρ*) above 0.4 was found, two factors still showed statistical significance (Table 3):

(1) Attitude to nursing was correlated with total scale (Spearman's *ρ* = 0.15, *P* < 0.001) and all the subscales positively (range of Spearman's *ρ* was from 0.12 to 0.15, *P* < 0.001), indicating that the better the attitude towards nursing, the higher the EBPQ score.
(2) Self-perceived working pressure was found to have positive correlations with attitude subscale (Spearman's *ρ* = 0.17, *P* < 0.001), showing that the higher the pressure, the lower the attitude score.

### 3.4. Comparisons of Groups of RNs

Depending on potential influential factors, RNs were classified into two (nursing administrator or not, research experience or not) or three (different working experience level) groups. We used nonparametric test to compare EBPQ of RNs with different clinical experience, while *t*-tests were applied to compare nursing administrator or not, as well as research experience or not (Table 3):

(1) Compared with RNs having administrative position, those without administrative position perceived lower scores not only in the total scale (*t* = −2.63, *P* < 0.01) but also in two subscales: attitude (*t* = −2.22, *P* < 0.05) and knowledge (*t* = −2.39, *P* < 0.05).
(2) Different clinical experience had statistical difference in scores of the knowledge subscale (*H* = 6.64, *P* < 0.05). After using Wilcoxon rank test, those with >15 years of clinical experience had higher scores compared with those with <10 years in the knowledge subscale. In addition, if we only compare the absolute values of each group, the values of >15-year clinical experience group were almost higher than the other two groups except for attitude subscale.
(3) RNs having research experience perceived higher scores not only in the total scale (*t* = −2.01, *P* < 0.05) but also in two subscales, attitude (*t* = −2.40, *P* < 0.05) and knowledge (*t* = −2.43, *P* < 0.05), than those without research experience.

## 4. Discussion

The results of this investigation indicate that our respondents generally viewed EBP positively and their attitudes towards EBP tended to be more positive than their knowledge/skills and practice of EBP. These results are in accordance with previous studies of a variety of professional healthcare groups [26–28]. That indicates that good RNs' EBP attitudes commonly do not equal good ability to implement EBP and this is not unique to RNs in China's TCM field. On the other hand, this enhances our confidence that we have obtained a relative reliable result.

Compared with the results of other recent similar studies conducted in other countries, we found that the average scores of our sample are lower in all the subscales [17, 29], especially lower than those in developed countries (United States and United Kingdom) [30]. That indicates there is a big gap between TCN field and nursing in western and middle eastern countries in EBP.

Our sample of RNs reported generally favorable attitudes towards EBP, with most agreeing that EBP is fundamental to professional practice. Noteworthy was that two-thirds positively agreed that there will be a “changing practice due to evidence found” and they “welcome questions on their practice,” whereas only a fourth positively agreed that they “make time in a work schedule for research.” These findings suggest that the RNs might have realized the importance of EBP and accepted the necessity for implementing it. However, lacking time and the higher working stress may make them hesitate when EBP is put into practice. The result of our previous study (lacking time and busier working were the greatest barrier of research utilization) also supports this point [24].

RNs in this survey reported that their poorest EBP skills were in “researching” and “critically analyzing evidence against set standards and retrieving evidence.” Given that this survey was of half of RNs with distance education which does not emphasize research training enough, this result is understandable. This also reflects a question: what is the main role of RNs during EBP? They are “user” of evidence in most of the time given their working characteristics. However, the academic faculty might be the best “producer” of evidence since a previous study found that they reported greater EBP knowledge/skills than clinical staff [30]. Therefore, cooperation between researchers in nursing school or academics, librarians, and RNs in hospitals should be encouraged [31]. On the other hand, only half of RNs got above-moderate scores in “disseminating new ideas about care to colleagues” and “sharing ideas and information with colleagues,” which is still a welcome result because dissemination is the indispensable part during EBP sustainability course [32].

Most of our sample reported poor practice in EBP, particularly in relation to “critically appraising literature,” “evaluating the outcomes of own practice,” and “sharing information with colleagues.” Only half of the respondents rated themselves with the positive scores in “formulating clear questions.” This indicates that RNs have a limited action of EBP. And a possible explanation might be that the introduction of EBP into the nursing field in China is relatively new now. Hence, the implementation status in the clinical setting is just a start.

In addition to examining EBP-related differences among those in different subgroups, our study highlighted several factors in relation to three aspects of EBP. First, better attitude towards nursing appears to be a good factor on all of the three EBP subscales. According to Namdar et al., attitude plays a principal role in guiding human behavior towards achieving goals [33]; therefore, nursing professional attitude affecting EBP is taken for granted. Second, RNs with research experience and administrative positions are better than those without either in attitude and knowledge. This is not surprising. On the one hand, nurse managers are always at the interface of policy and practice and they should translate the demand of the hospitals into meaningful and feasible tasks for the nurses. EBP could appear as a solution to such difficult translation just in time. Hence, nurse managers welcome EBP with open arms. And, actually, nursing managerial leadership could really push the EBP progress [34]. On the other hand, the core of EBP is to recognize, to appraise, and to apply the best available research findings (lines of evidence). That might be the reason why RNs with research experience enjoyed better performance in our survey. Finally, our data also showed that RNs with more years of experience reported stronger knowledge and RNs under lighter working stress perceived more positive attitude towards EBP.

It is worthy to note that our previous analysis on the barriers of RU also found several same influential factors like those of knowledge/skills, attitude, and practice of EBP. That implies that barriers of RU might have some certain relations with knowledge/skills, attitude, and practice of EBP. We found that several items are similar, but only in the knowledge EBPQ subscale. It is because of similar influential factors found that we could not avoid drawing some similar implications from two articles. After deep consideration, we have to admit that using these two instruments together might cause some overlap in our survey. Hence, we suggest that researchers who keep watchful eyes on this area should consider our flaw in their future research. On the other hand, one factor, administrative role or not, was the new finding. The possible reason might be that all RNs were faced with the same level of barriers, but RNs with administrative position hold stronger attitude and knowledge/skills on EBP.

Based on our knowledge, the present study, which examined the EBP attitude, knowledge, and practice of RNs, is the first one in China's TCM hospitals. And this survey was based on four TCM hospitals with a sample of 648 RNs and applied international standard questionnaire. However, the results should be viewed cautiously, taking into account some limitations. First, the sample was not randomized, which may not be truly representative of the RNs in TCM field. But we tried to take cluster samples as much as possible in order to minimize potential bias. Second, all the data was reported by RNs which might cause inherent bias.

Taken together, we proposed several suggestions for the clinical practice and future research. First, cooperation between academic faculty and clinical RNs should be encouraged. A working chain, “producer/provider (faculty)-user (RNs)-researcher (faculty and RNs),” might be a choice to complement each other's advantages. Furthermore, it is best for the EBP team to contain RNs with longer working experience. Second, reducing RNs' working loads or increasing nurses should be considered if the hospital intends to carry out EBP in routine nursing work. Third, our data support the importance of providing advanced education and training course for nurses to facilitate the EBP, which is consistent with the findings of other studies [35]. Finally, due to the mentioned limitations of our sample, a random sampling, multiple-level hospitals designed survey is needed.

## 5. Conclusions

By means of analyzing 648 RNs from four TCM hospitals in China, we evaluated the status of implementation of EBP in daily TCN clinical practice. Although the three aspects of EBP of our respondents are not as positive as those in other countries, we estimate that our respondents generally viewed EBP positively and their attitudes towards EBP tended to be more positive than their knowledge/skills and practice of EBP. Our data also showed that several factors, longer working experience, having administrative position, research experience, lighter working load, and better professional attitude, might facilitate EBP. To the possible extent, education and training that support these factors may help to increase positive beliefs and attitudes regarding EBP and, ultimately, EBP use in practice.

## Figures and Tables

**Table 1:** Comparison means and standard deviations of the EBPQ and its subscales with recent similar studies in other countries (*N* = 648).

Scale or subscales	Our survey 2015	95% CI	China 2007	USA 2014	UK 2014	Spain 2014	Iran 2014
EBPQ total	4.24 ± 0.79	4.18~4.30	4.24 ± 0.79	Not reported	Not reported	4.81 ± 1.09^∧^	4.48 ± 1.26^∧^
Practice	4.08 ± 1.05	4.00~4.16	3.99 ± 0.97^*∗*^	5.00	5.67	4.59 ± 1.53^∧^	4.58 ± 1.24^∧^
Knowledge/skills	4.17 ± 0.84	4.10~4.23	4.25 ± 0.83^*∗*^	5.71	6.00	4.56 ± 1.15^∧^	4.39 ± 1.20^∧^
Attitude	4.75 ± 1.01	4.67~4.82	4.56 ± 1.06^∧^	5.75	5.25	5.28 ± 1.10^∧^	4.57 ± 1.35^∧^

^*∗*^ *P* < 0.05;  ^∧^ *P* < 0.001.

In Spain 2014, we only extracted the data related to RNs.

**Table 2:** Scores of each EBPQ item (*N* = 648).

Item	Score (mean ± SD)	Response patterns % responding 1–4^*∗*^	Priority item rank
*Practice*			
Critically appraising literature	(3.76 ± 1.27)	75.2	1
Evaluating the outcomes of own practice	(3.76 ± 1.40)	72.2	2
Sharing information with colleagues	(3.98 ± 1.54)	64.7	3
Integrating evidence with expertise	(4.11 ± 1.33)	63.9	4
Finding relevant evidence	(4.30 ± 1.25)	61.1	5
Formulating clear questions	(4.58 ± 1.11)	52.3	6
*Attitudes*			
Making time in a work schedule for research	(3.84 ± 1.29)	72.8	1
Welcoming questions on own practice	(4.97 ± 1.31)	40.1	2
Changing practice due to evidence found	(5.09 ± 1.26)	35.2	3
EBP is fundamental to professional practice	(5.07 ± 1.28)	34	4
*Knowledge/skills*			
Research skills	(3.97 ± 1.17)	83.8	1
Critically analyzing evidence against set standards	(3.79 ± 1.13)	75.3	2
Retrieving evidence	(4.01 ± 1.01)	70.5	3
Determining the validity (close to the truth) of material	(4.05 ± 1.05)	69.9	4
Converting information needs into a research question	(3.76 ± 1.27)	68.4	5
Awareness of major information types/sources	(4.11 ± 1.08)	65.7	6
Determining how useful (clinically applicable) material is	(4.18 ± 1.01)	63.9	7
IT skills	(4.20 ± 1.10)	63.1	8
Monitoring and reviewing practice	(4.28 ± 1.00)	61.7	9
Applying information to individual cases	(4.28 ± 1.10)	60.8	10
Identifying gaps in professional practice	(4.35 ± 0.96)	58.2	11
Reviewing own practices	(3.76 ± 1.27)	52.5	12
Disseminating new ideas about care to colleagues	(4.58 ± 1.19)	50.5	13
Sharing ideas and information with colleagues	(4.61 ± 1.16)	48.1	14

^*∗*^1 means never/poor; 7 means always/best.

**Table 3:** Correlations between the nurses' demographic variables and their scores on the EBPQ by subscale (*N* = 414).

Characteristics	Questionnaire mean score ± SD			
	Practice	Attitudes	Knowledge/skills	Total EBPQ
Clinical experience				
<10 years	4.03 ± 1.05	4.78 ± 1.04	4.13 ± 0.86	4.21 ± 0.81
10~15 years	4.09 ± 1.00	4.67 ± 0.86	4.12 ± 0.82	4.21 ± 0.74
>15 years	4.31 ± 1.05	4.69 ± 1.02	4.37 ± 0.79	4.41 ± 0.74
*P *value	0.115	0.469	0.036	0.078
Nursing administrator				
Yes	4.33 (1.18)	5.00 (0.96)	4.40 (0.86)	4.48 (0.82)
No	4.05 (1.03)	4.72 (1.01)	4.14 (0.84)	4.22 (09.78)
*P *value	0.075	0.023	0.022	0.009
Attitude to nursing				
Like	4.34 ± 1.06	5.13 ± 1.04	4.36 ± 0.92	4.49 ± 0.86
Neither like nor dislike	4.13 ± 1.02	4.77 ± 1.00	4.23 ± 0.82	4.49 ± 0.86
Dislike	3.88 ± 1.06	4.54 ± 0.97	3.95 ± 0.82	4.03 ± 0.75
*P* value	0.001	<0.001	<0.001	<0.001
Research experience				
Yes	4.08 ± 1.05	4.86 ± 0.93	4.26 ± 0.86	4.32 ± 0.81
No	4.08 ± 1.04	4.67 ± 1.06	4.10 ± 0.82	4.19 ± 0.77
*P* value	0.710	0.027	0.012	0.038
*Self-perceived* working pressure				
No pressure at all	4.33	4.25	3.57	3.88
A little	5.33	5	4.29	4.67
Just so-so	4.10 ± 1.08	5.01 ± 0.99	4.28 ± 0.85	4.36 ± 0.81
Moderate	4.05 ± 1.00	4.68 ± 0.97	4.10 ± 0.80	4.18 ± 0.75
Great pressure	4.14 ± 1.12	4.54 ± 1.08	4.18 ± 0.92	4.23 ± 0.84
*P* value	0.696	<0.001	0.081	0.127

*Note.* SD: standard deviation.
